# Supplementary figures and images for: Senataxin and RNase H2 act redundantly to suppress genome instability during class switch recombination
Source: eLife. 2022 Dec 21;11:e78917. doi: 10.7554/eLife.78917 (PMC9771370; doi:10.7554/eLife.78917)

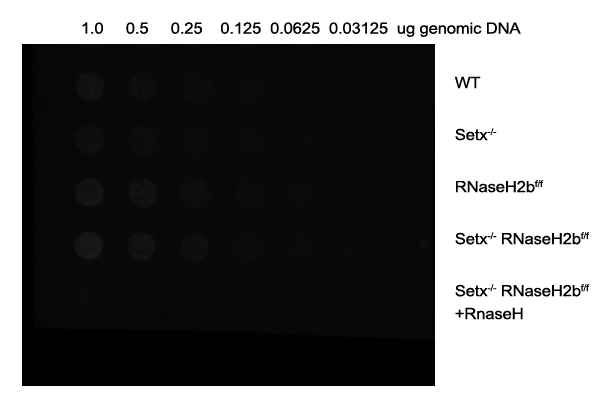

Supplement: Figure 1—source data 1. — Genomic DNA extracted from WT, Rnaseh2bf/f, Setx-/-, and Setx-/-Rnaseh2bf/f cells was digested with restriction enzyme cocktail and run on dot blot, RnaseH1-treated Setx-/-Rnaseh2bf/f as a negative control. [file elife-78917-fig1-data1.zip › Figure 1B- dot blot source data 2.tif]

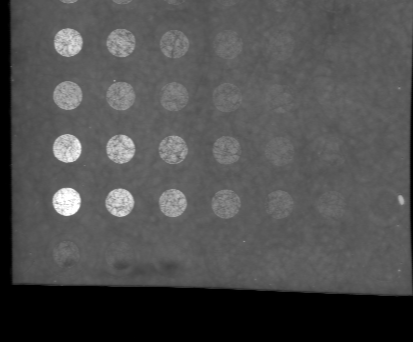

Supplement: Figure 1—source data 1. — Genomic DNA extracted from WT, Rnaseh2bf/f, Setx-/-, and Setx-/-Rnaseh2bf/f cells was digested with restriction enzyme cocktail and run on dot blot, RnaseH1-treated Setx-/-Rnaseh2bf/f as a negative control. [file elife-78917-fig1-data1.zip › Figure 1B- dot blot source data 1.tif]

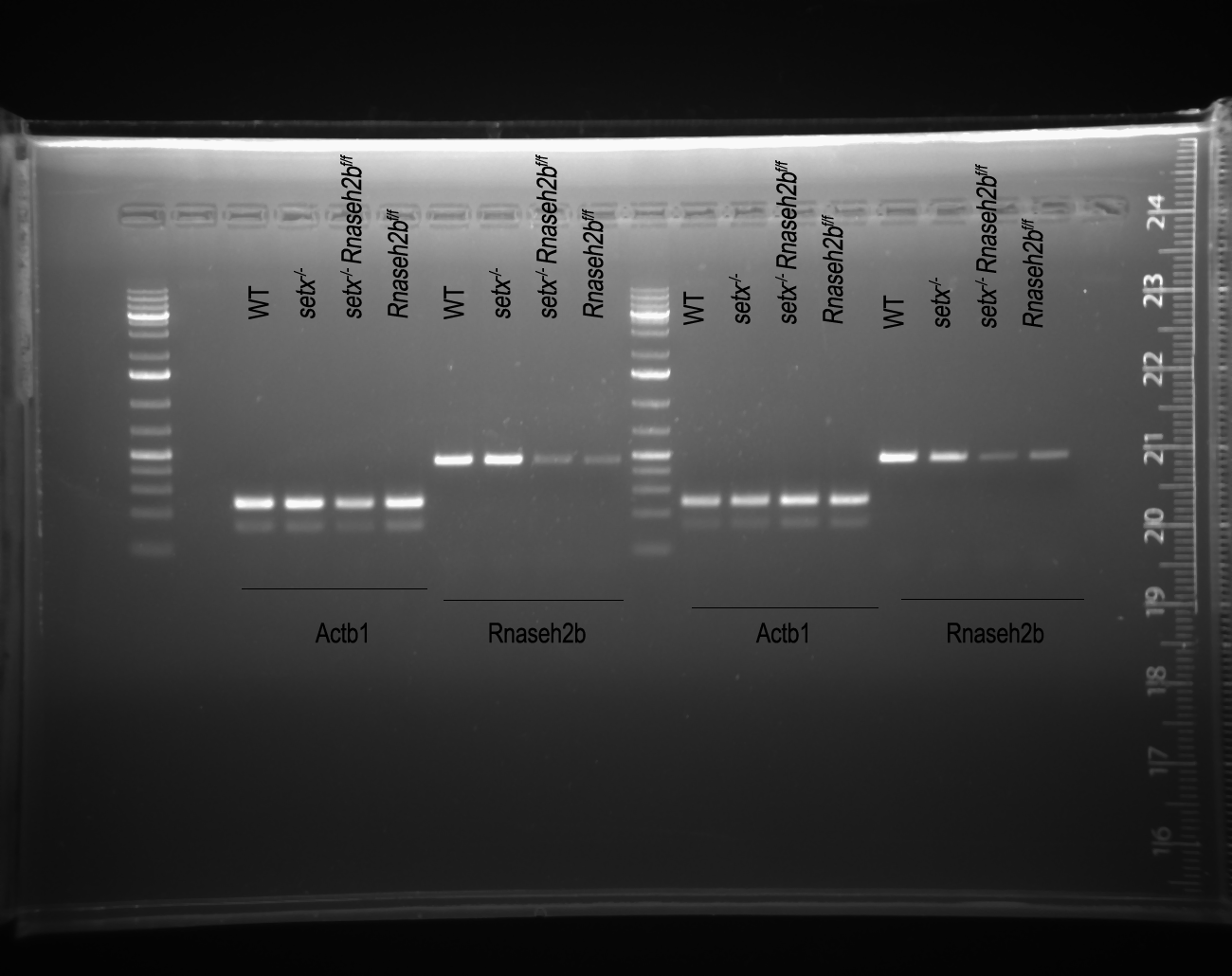

Supplement: Figure 1—figure supplement 1—source data 1. — PCR test of Rnaseh2b deletion efficiency on genomic DNA extracted from WT, Setx-/-, Rnaseh2bf/f, and Setx-/- Rnaseh2bf/f splenic B lymphocytes. Left and right represent two times results. [file elife-78917-fig1-figsupp1-data1.zip › Figure 1 - figure supplement 1A 2B gel source 2.tif]

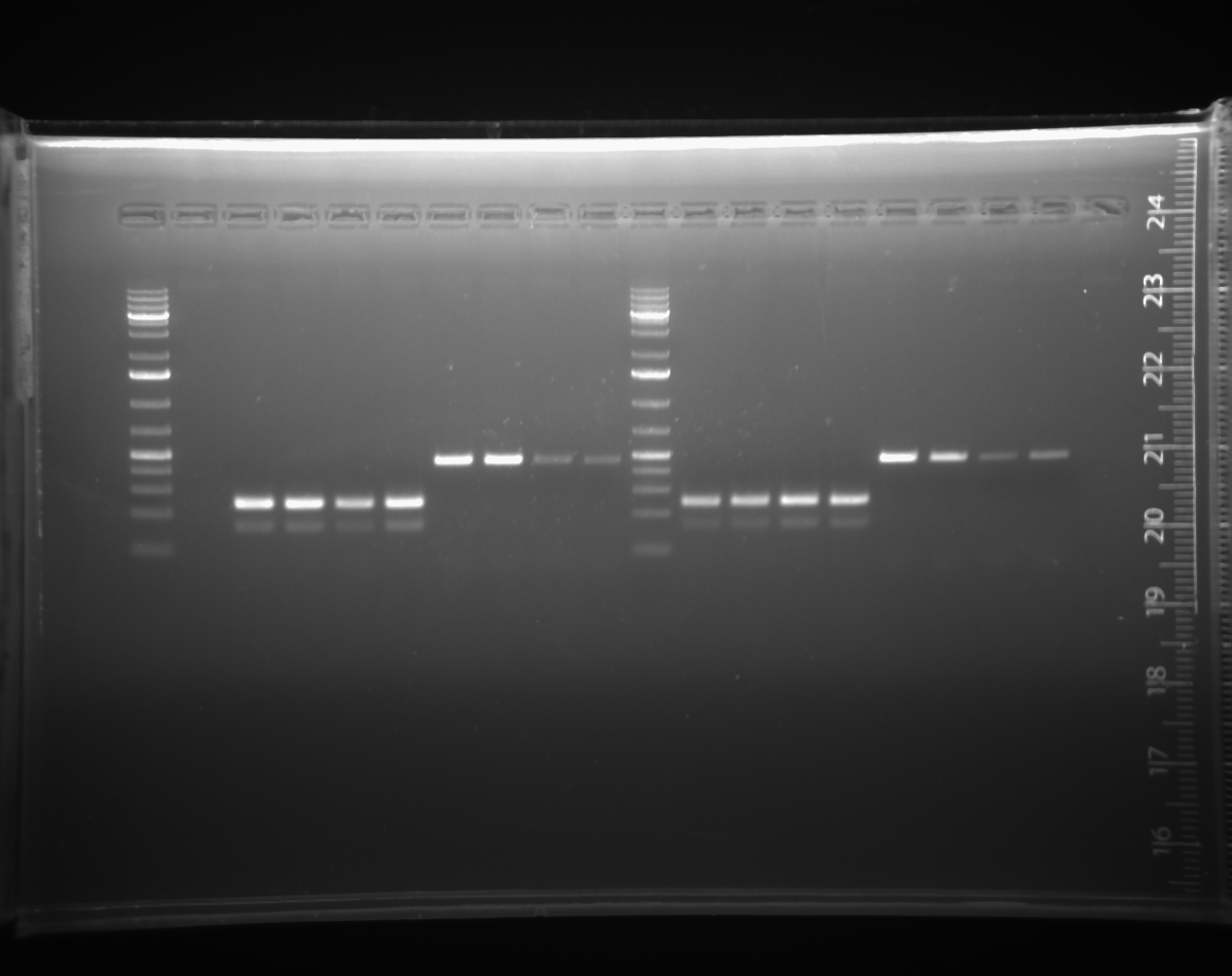

Supplement: Figure 1—figure supplement 1—source data 1. — PCR test of Rnaseh2b deletion efficiency on genomic DNA extracted from WT, Setx-/-, Rnaseh2bf/f, and Setx-/- Rnaseh2bf/f splenic B lymphocytes. Left and right represent two times results. [file elife-78917-fig1-figsupp1-data1.zip › Figure 1 - figure supplement 1A 2B gel source 1.tif]

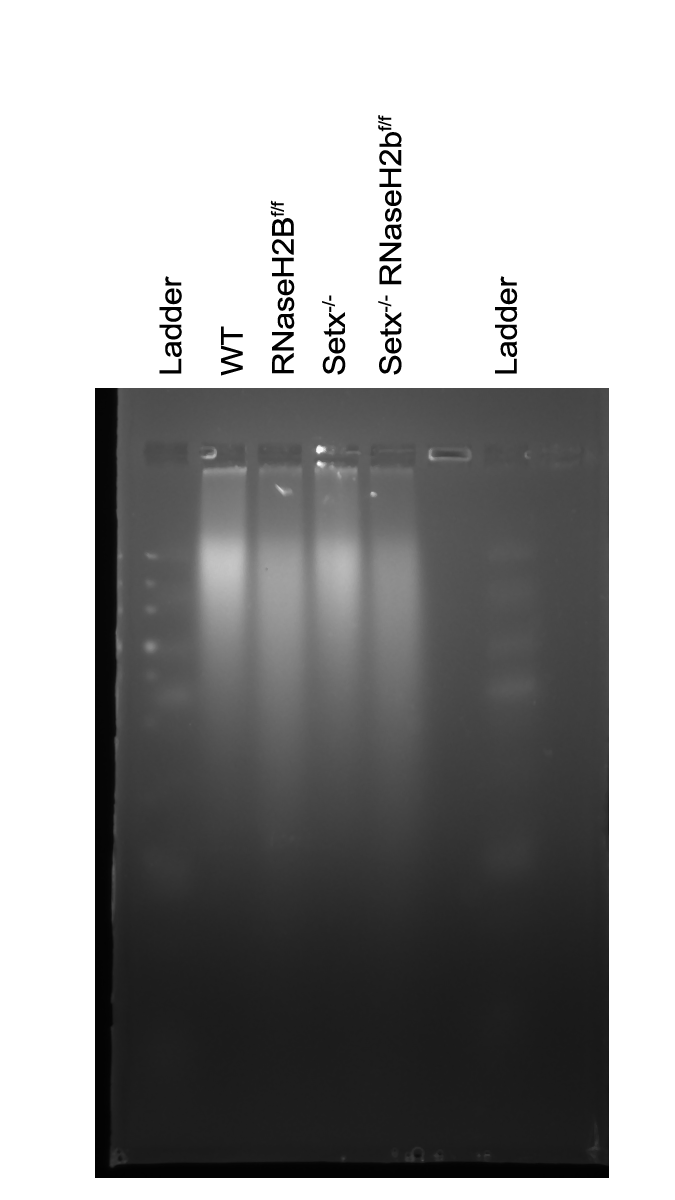

Supplement: Figure 3—figure supplement 2—source data 1. — Genomic DNA from WT, Rnaseh2bf/f, Setx-/-, and Setx-/-Rnaseh2bf/f B cells was treated with NaOH for 3 hr before running the alkaline gel. [file elife-78917-fig3-figsupp2-data1.zip › Figure 3 - figure supplement 2A source data 2.tif]

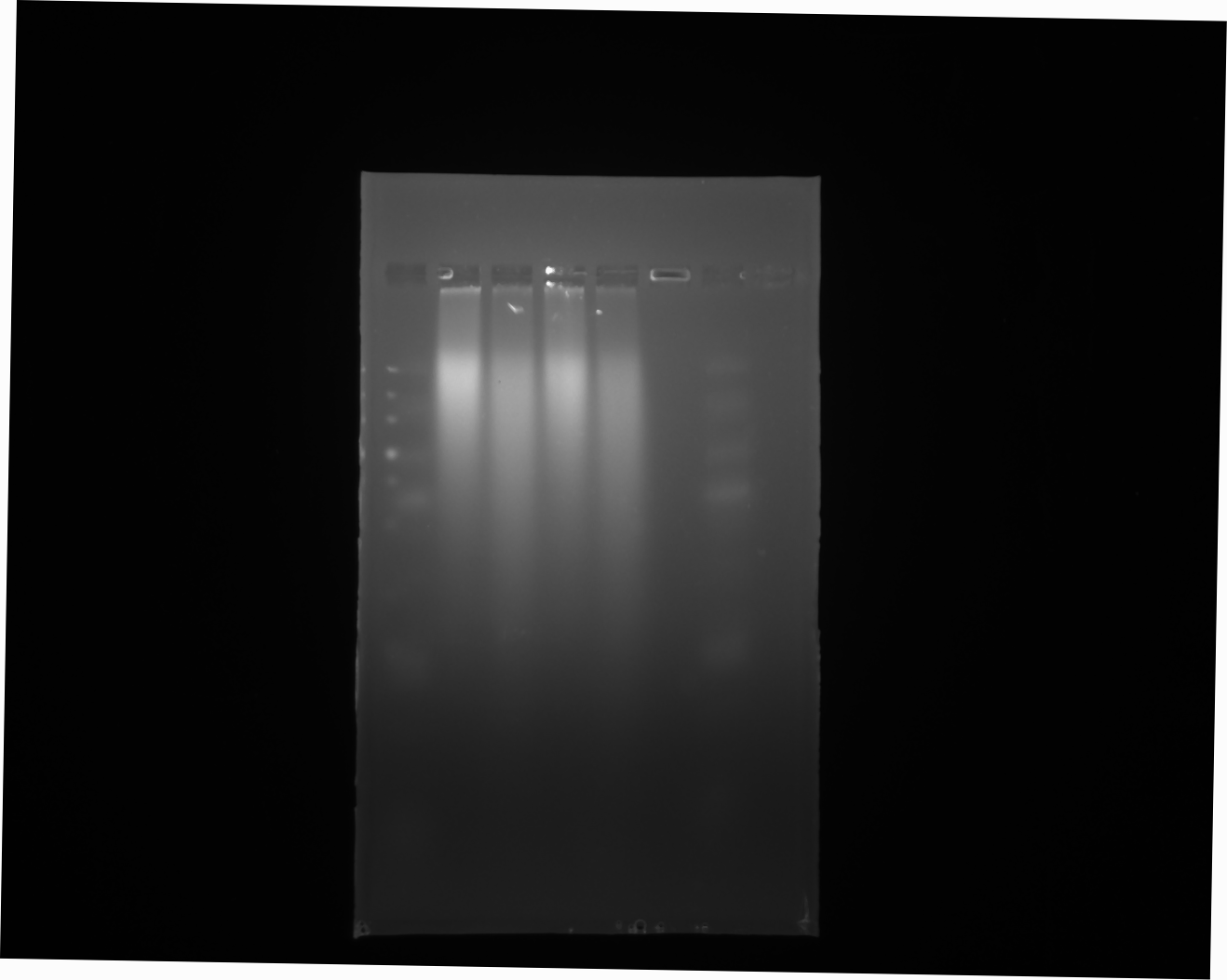

Supplement: Figure 3—figure supplement 2—source data 1. — Genomic DNA from WT, Rnaseh2bf/f, Setx-/-, and Setx-/-Rnaseh2bf/f B cells was treated with NaOH for 3 hr before running the alkaline gel. [file elife-78917-fig3-figsupp2-data1.zip › Figure 3 - figure supplement 2A source data 1.tif]

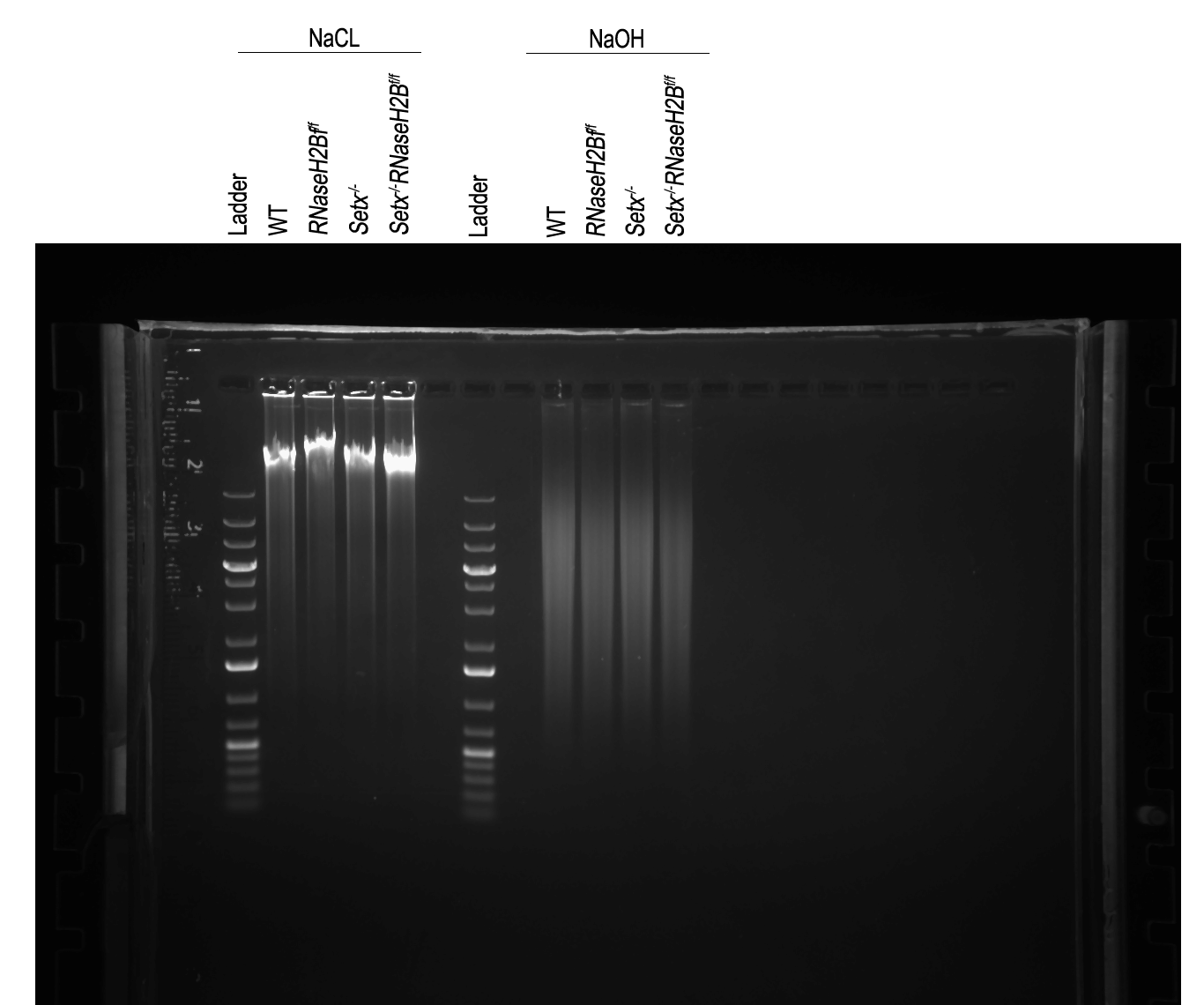

Supplement: Figure 3—figure supplement 2—source data 2. — Genomic DNA from WT, Rnaseh2bf/f, Setx-/-, and Setx-/-Rnaseh2bf/f B cells was treated with NaCl (left) or NaOH (right) for 3 hr before running the TAE native gel. [file elife-78917-fig3-figsupp2-data2.zip › Figure 3 - figure supplement 2C native gel source data 2.tif]

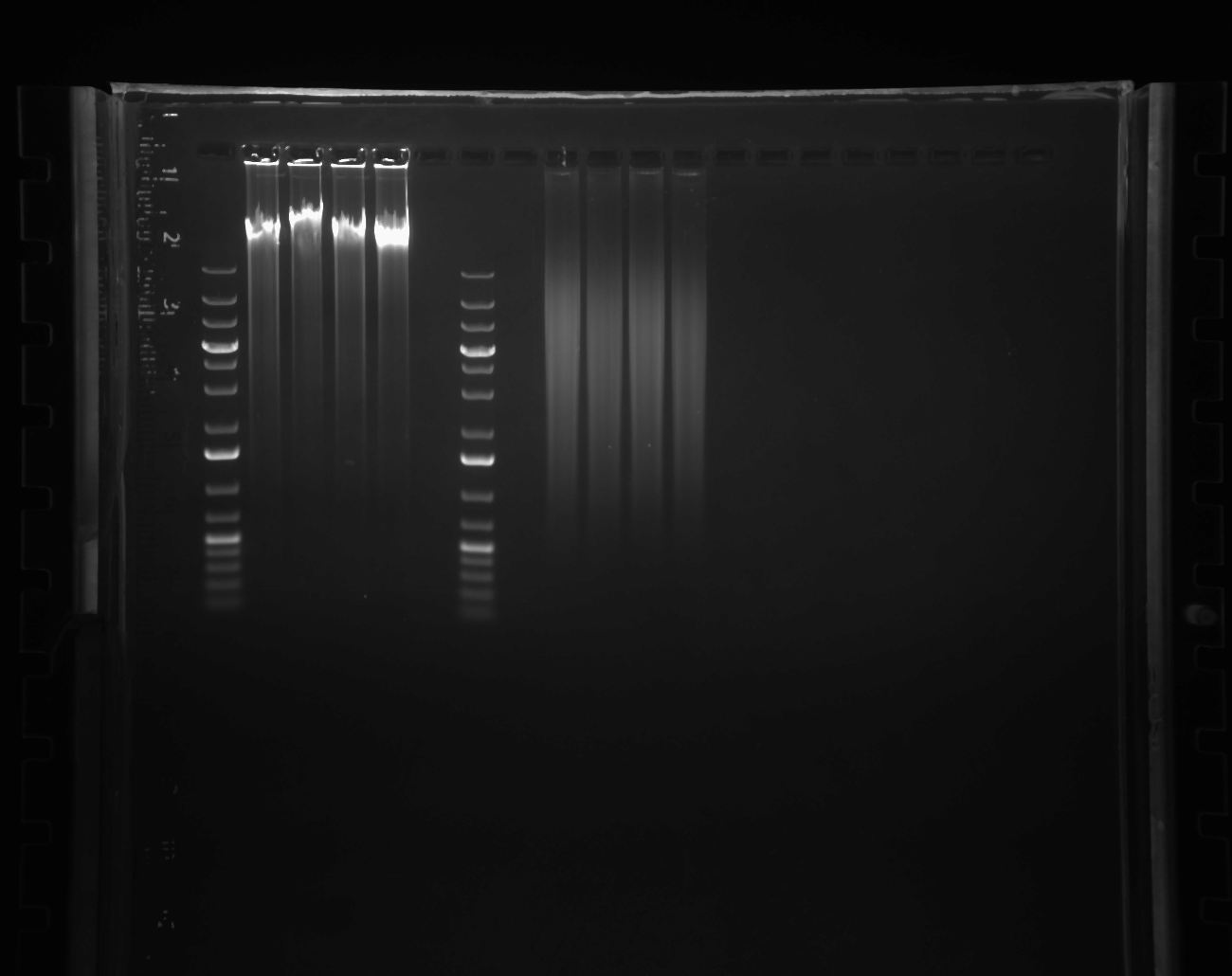

Supplement: Figure 3—figure supplement 2—source data 2. — Genomic DNA from WT, Rnaseh2bf/f, Setx-/-, and Setx-/-Rnaseh2bf/f B cells was treated with NaCl (left) or NaOH (right) for 3 hr before running the TAE native gel. [file elife-78917-fig3-figsupp2-data2.zip › Figure 3 - figure supplement 2C native gel source data 1.tif]

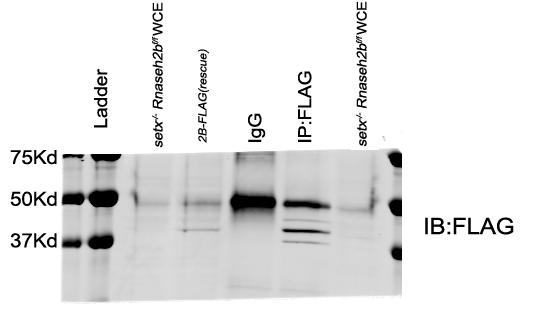

Supplement: Figure 4—source data 1. — Immunoprecipitation and immunoblotting test for RNaseH2B-FLAG protein expression under retroviral infection in Setx-/-Rnaseh2bf/f B cells. [file elife-78917-fig4-data1.zip › Figure 4A-2b rescue Source Data 2.tif]

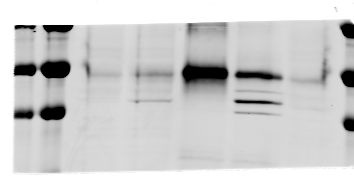

Supplement: Figure 4—source data 1. — Immunoprecipitation and immunoblotting test for RNaseH2B-FLAG protein expression under retroviral infection in Setx-/-Rnaseh2bf/f B cells. [file elife-78917-fig4-data1.zip › Figure 4A-2b rescue Source Data 1.TIF]

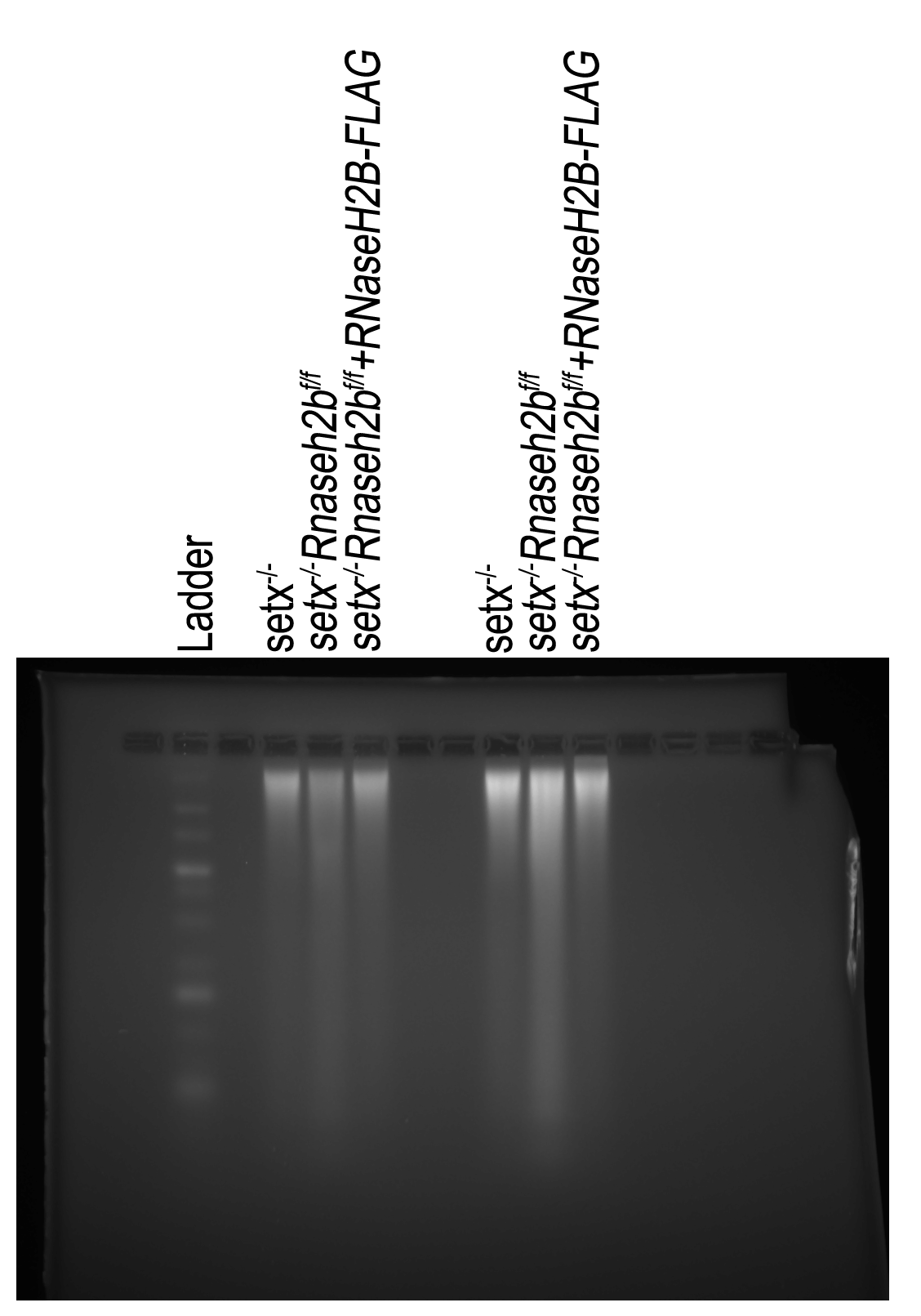

Supplement: Figure 4—source data 2. — Uncropped image of alkaline gel from Setx-/-, Setx-/-Rnaseh2bf/f EV, and Setx-/-Rnaseh2bf/f+FLAG-RNaseH2B cells; left and right represent two times results. [file elife-78917-fig4-data2.zip › Figure 4B source data 2.tif]

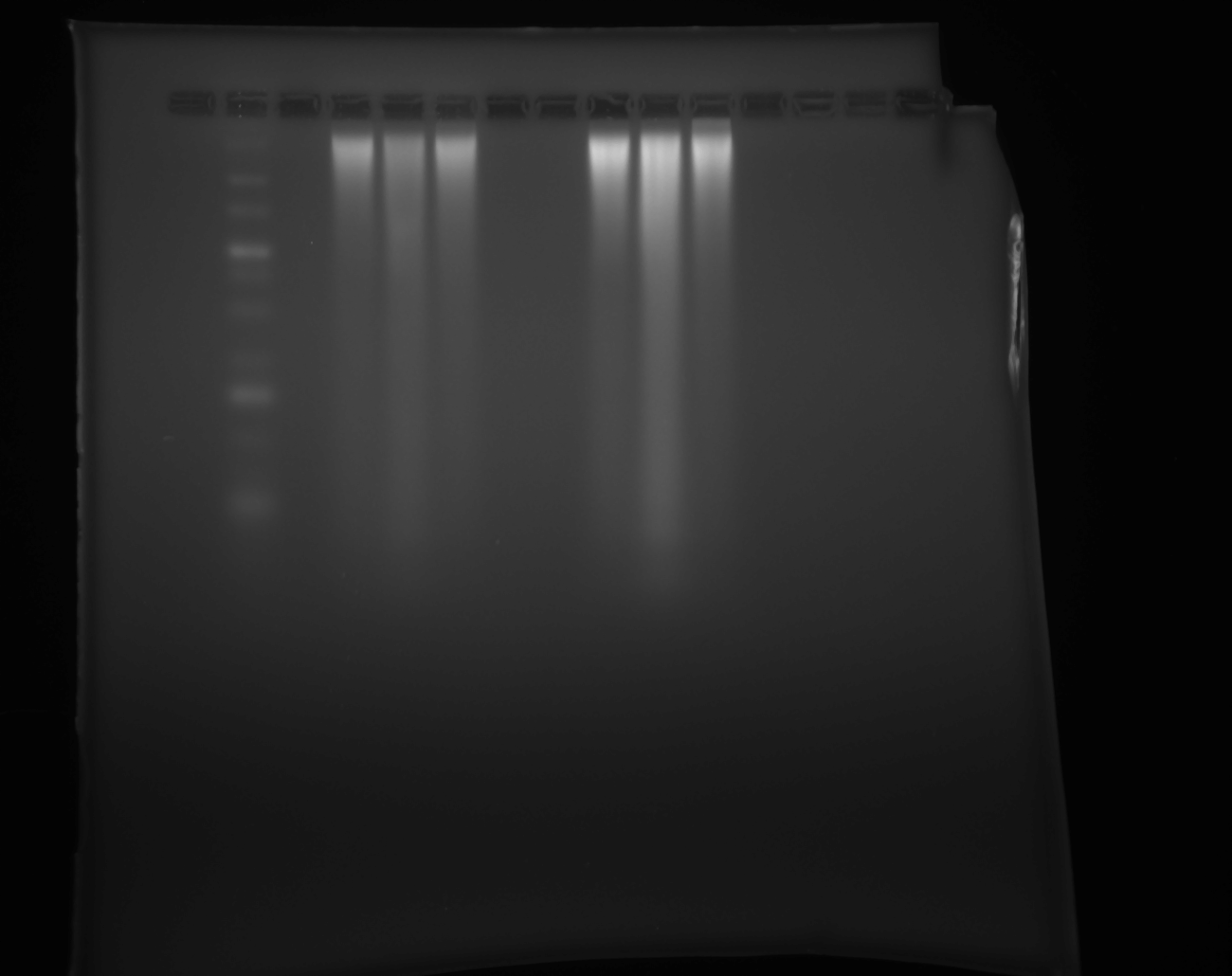

Supplement: Figure 4—source data 2. — Uncropped image of alkaline gel from Setx-/-, Setx-/-Rnaseh2bf/f EV, and Setx-/-Rnaseh2bf/f+FLAG-RNaseH2B cells; left and right represent two times results. [file elife-78917-fig4-data2.zip › Figure 4B source data 1.tif]

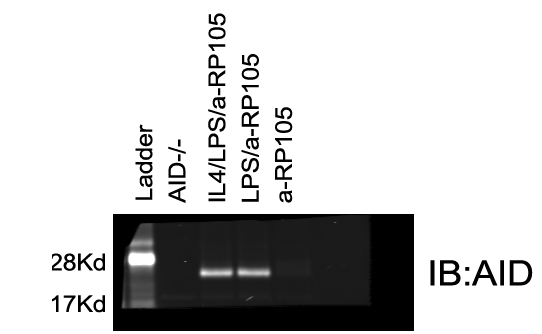

Supplement: Figure 5—source data 1. — AID protein expression in WT B cells 72 hr post-stimulation with different reagents. Actin served as a loading control. [file elife-78917-fig5-data1.zip › Figure 5A-AID Source Data 2.tif]

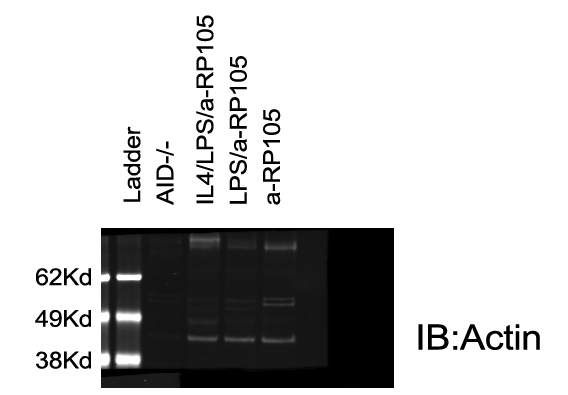

Supplement: Figure 5—source data 1. — AID protein expression in WT B cells 72 hr post-stimulation with different reagents. Actin served as a loading control. [file elife-78917-fig5-data1.zip › Figure 5A-Actin Source Data 2.tif]

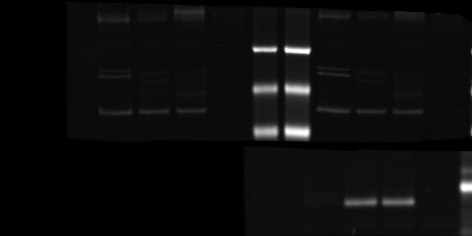

Supplement: Figure 5—source data 1. — AID protein expression in WT B cells 72 hr post-stimulation with different reagents. Actin served as a loading control. [file elife-78917-fig5-data1.zip › Figure 5A AID and Actin Souce data 1.TIF]

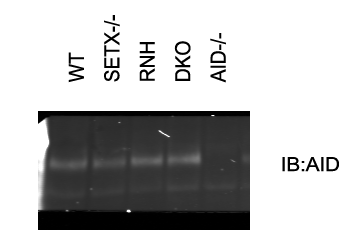

Supplement: Figure 5—figure supplement 1—source data 1. — Whole-cell lysis from WT, Setx-/-, Rnaseh2bf/f, Setx-/-Rnaseh2bf/f, and Aicda-/- cells 72 hr post-stimulation to IgG1 were run on SDS-PAGE. Western blots were probed with an anti-AID antibody and anti-actin as loading control. Aicda-/- cells were used as a negative control. [file elife-78917-fig5-figsupp1-data1.zip › Figure 5 - figure supplement 1B-AID Source Data 2.tif]

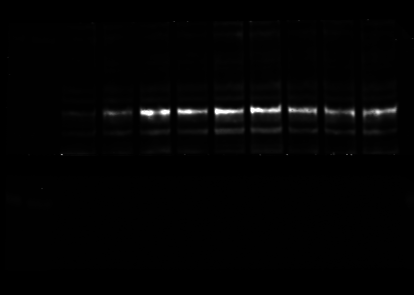

Supplement: Figure 5—figure supplement 1—source data 1. — Whole-cell lysis from WT, Setx-/-, Rnaseh2bf/f, Setx-/-Rnaseh2bf/f, and Aicda-/- cells 72 hr post-stimulation to IgG1 were run on SDS-PAGE. Western blots were probed with an anti-AID antibody and anti-actin as loading control. Aicda-/- cells were used as a negative control. [file elife-78917-fig5-figsupp1-data1.zip › Figure 5 - figure supplement 1B-actin Source Data 1.tif]

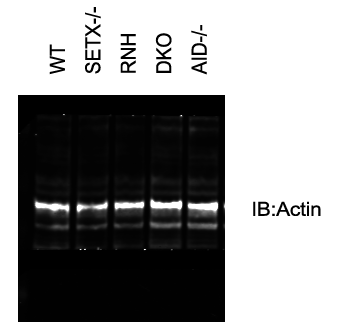

Supplement: Figure 5—figure supplement 1—source data 1. — Whole-cell lysis from WT, Setx-/-, Rnaseh2bf/f, Setx-/-Rnaseh2bf/f, and Aicda-/- cells 72 hr post-stimulation to IgG1 were run on SDS-PAGE. Western blots were probed with an anti-AID antibody and anti-actin as loading control. Aicda-/- cells were used as a negative control. [file elife-78917-fig5-figsupp1-data1.zip › Figure 5 - figure supplement 1B-actin Source Data 2.tif]

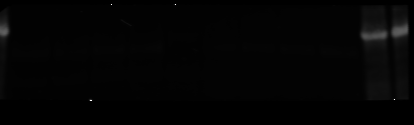

Supplement: Figure 5—figure supplement 1—source data 1. — Whole-cell lysis from WT, Setx-/-, Rnaseh2bf/f, Setx-/-Rnaseh2bf/f, and Aicda-/- cells 72 hr post-stimulation to IgG1 were run on SDS-PAGE. Western blots were probed with an anti-AID antibody and anti-actin as loading control. Aicda-/- cells were used as a negative control. [file elife-78917-fig5-figsupp1-data1.zip › Figure 5 - figure supplement 1B-AID Source Data 1.tif]
